# Islam and family planning: changing perceptions of health care providers and medical faculty in Pakistan

**DOI:** 10.9745/GHSP-D-13-00019

**Published:** 2013-06-26

**Authors:** Ali Mohammad Mir, Gul Rashida Shaikh

**Affiliations:** a Population Council, Islamabad, Pakistan

## Abstract

A USAID-sponsored family planning project called “FALAH” (Family Advancement for Life and Health), implemented in 20 districts of Pakistan, aimed to lower unmet need for family planning by improving access to services. To enhance the quality of care offered by the public health system, the FALAH project trained 10,534 facility-based health care providers, managers, and medical college faculty members to offer client-centered family planning services, which included a module to explain the Islamic viewpoint on family planning developed through an iterative process involving religious scholars and public health experts. At the end of the FALAH project, we conducted a situation analysis of health facilities including interviews with providers to measure family planning knowledge of trained and untrained providers; interviewed faculty to obtain their feedback about the training module; and measured changes in women's contraceptive use through baseline and endline surveys. Trained providers had a better understanding of family planning concepts than untrained providers. In addition, discussions with trained providers indicated that the training module on Islam and family planning helped them to become advocates for family planning. Faculty indicated that the module enhanced their confidence about the topic of family planning and Islam, making it easier to introduce and discuss the issue with their students. Over the 3.5-year project period, which included several components in addition to the training activity, we found an overall increase of 9 percentage points in contraceptive prevalence in the project implementation districts—from 29% to 38%. The Islam and family planning module has now been included in the teaching program of major public-sector medical universities and the Regional Training Institutes of the Population Welfare Department. Other countries with sizeable Muslim populations and low contraceptive prevalence could benefit from this module.

## Abstract (precis)

Training health care providers and medical college faculty about the supportive nature of Islam toward family planning principles addressed their misconceptions and enhanced their level of comfort in providing family planning services and teaching the subject.

## BACKGROUND

Pakistan, currently the sixth most populous country in the world, was a pioneer in the region by launching a full-fledged family planning program in the mid-1960s. However, the program has achieved only modest success. Contraceptive prevalence has stagnated at 30%—the lowest level among neighboring countries.^1^ Use of modern contraceptive methods to delay or limit pregnancy is 22%. The most widely used modern method is female sterilization (8%) while use of long-acting reversible contraceptives is at about 2% (mostly from use of IUDs). About one-third of births occur within birth intervals of less than 24 months.^1^ One-quarter of women of reproductive age want to space or limit their births but are not using contraception and therefore have an unmet need.^1^ This is mainly due to issues related to access to services, restrictions on women's mobility outside their homes,^2^ fear of side effects of modern contraceptives, and perceived social disapproval from their spouses or other family members,^3^ partly attributed to misconceptions about the permissibility of family planning in Islam.

The majority (97%) of Pakistan's population is Muslim.^1^ The influence of religion is pervasive in all aspects of an individual's life, including personal matters such as managing family size. A study conducted by the Pakistan National Institute of Population Studies showed that women in communities where *Ulema* (Muslim religious leaders) gave permission to use birth spacing methods were 1.7 times more likely to use contraceptives than women in communities where *Ulema* did not allow family planning programs (Odds Ratio [OR] = 1.704, 95% confidence interval [CI] = 1.11–2.6; *P* = 0.014).^4^ A qualitative study in rural Pakistan found that men often cited religion as an important reason for not using contraception,^5^ and the Pakistan Demographic and Health Survey reported that 5% of married women do not use contraception due to religious reasons.^1^

Religious opposition sometimes can be a deterrent to contraceptive use.

Although there has never been any vocal opposition to family planning by religious conservatives in Pakistan, successive governments fearful of a backlash have been cautious in involving religious leaders in family planning activities. This lack of engagement has led to ambiguity in the minds of the public about the acceptability of family planning in Islam.

The rationale for family planning propagated over the years focused primarily on reducing family size to alleviate demographic and economic issues facing the country, which many saw as an infringement by the state to their personal decision-making freedom. Thus, individuals failed to appreciate the direct benefits of family planning on their lives. Information on the health benefits of family planning, which could have influenced the stance of religious leaders, was unfortunately not available to them. It was not until 2006 that a conference of religious leaders was organized to develop religious consensus on the issue.

Between 2007 and 2012, the Population Council in Pakistan, leading a consortium of 7 organizations, implemented a family planning project called “FALAH” (Family Advancement for Life and Health), with funding from the U.S. Agency for International Development (USAID). The project aimed to reduce unmet need for family planning by improving access to services in 20 districts of the country.

We repositioned family planning as a health intervention, introducing the concept of “birth spacing saves lives” based on the principles of healthy timing and spacing of pregnancies (HTSP) to reduce maternal, newborn, and child mortality and morbidity. To accomplish this, we involved the Department of Health more fully. One obvious advantage of doing so was that the Department of Health has a much larger infrastructure with 14,287 facilities compared with the 2,891 family welfare centers of the Population Welfare Department. We worked to improve the quality of care offered by public-sector facilities of both departments by ensuring that they were well-stocked with an uninterrupted supply of contraceptives and by training providers to offer client-centered family planning services.

A key component of the FALAH project was a behavior change training program for providers and medical college faculty that included modules on (see supplementary material):

- Instilling a sense of self-worth among the providers and encouraging them to view their work as a social responsibility
- How to communicate with clients on issues related to gender norms and power dynamics at the household level
- Use of the SAHR framework (Salutation, Assessment, Help, Reassurance) for holistically assessing and meeting clients' needs through mutual negotiations
- Enhancing providers' knowledge and skills on contraceptive methods, developed in collaboration with Jhpiego, a technical partner on the FALAH project

We also developed a module on “The Islamic Viewpoint on Family Planning” (see supplementary material). In this paper, we describe the rationale and process for developing this particular module. We also include feedback from interviews with faculty and providers about the impact of the training module on their teaching and practice, respectively, as well as results of the FALAH project overall on contraceptive use.

## INTERVENTION Description

### Needs Assessment

We first undertook an extensive needs assessment of medical institutions and colleges to identify areas that needed strengthening in teaching family planning. Our discussions with university vice chancellors and faculty members revealed that, due to a variety of reasons, they did not discuss family planning with their students thoroughly. Among the many weak areas identified, the one highlighted most often was faculty's concern about whether family planning was allowed in Islam.

For this reason, we developed 2 sets of training programs to deliver client-centered family planning services: one to train existing health care providers and the other to train faculty of medical colleges. Both training programs included the module on Islam and family planning.

### Obtaining Religious Consensus

In 2009, in collaboration with the Ministry of Population Welfare, we sponsored a national seminar entitled “Involvement of Religious Leaders in the Population Welfare Program.” We brought together noted Muslim scholars from the 2 major sects in Islam (Sunni and Shia) and the major subsects. The majority of Muslims in the country follow the Sunni Hanafi School of jurisprudence. The Sunnis are further divided into two subsects, the Barelvis and Deobandis.

During the seminar, we deliberated in groups with the scholars about the close scientific link between birth spacing, family size, and maternal and infant health outcomes, especially the relation between birth intervals and infant mortality based on figures available in the 2006–07 Pakistan Demographic and Health Survey and on international evidence.^6^-^9^ The seminar culminated in a consensus report in which the scholars unequivocally agreed that using modern and traditional contraceptive methods to space births was in no way contrary to Islamic teachings, allowing individuals to freely decide upon the number and timing of their children.^10^ We later also captured the scholars' views in a short video documentary (in Urdu with English subtitles) entitled *Farishton Ne Bhi Poocha* (Even the Angels Dared to Ask) (see http://www.youtube.com/watch?v = GBRtZTlZdHU).

Muslim scholars in Pakistan developed consensus on the permissibility of family planning in Islam.

### Review of Existing Curricula

After the seminar, we reviewed existing curricula and materials on the subject developed in other countries to explore the arguments and explanations that established the permissibility of family planning in Islam.^11^-^14^ We also studied edicts passed on the subject by the Grand Muftis (chief jurists who interpret Muslim law) of Jordan and Egypt.

After this review, we made a conscious decision to focus primarily on the Quran (the sacred text of Islam) because we believed that people's misconceptions had resulted from an inability to correctly collate and interpret the various verses of the Quran that deal with family responsibility and wellbeing. For instance, some Muslim religious leaders have interpreted that, although family planning is not explicitly prohibited in the Quran, it is not implicitly allowed—that is, that the Quran is silent on the issue.^15^ However, an in-depth study of Quranic injunctions reveals that this is not the case.

We also thought that focusing on Quranic teachings would be the most direct approach and difficult for people to contradict, therefore preventing any counterarguments and controversy.

### Introducing the Module

The FALAH project trained 10,534 facility-based health care providers, faculty members of the affiliated colleges of 6 medical universities, and managers and trainers of training institutes of the Population Welfare Department in the 20 project districts.

For the providers' training program, we developed master trainers from the Departments of Health and Population Welfare. These master trainers conducted the step-down trainings in batches consisting of 20 participants each. We oversaw the first iteration of the step-down trainings. For quality assurance, we made periodic visits to the training sites and provided any necessary feedback to the trainers using a training checklist.

For the faculty training program, the training team directly trained faculty members from the departments of Anatomy, Physiology, Community Medicine, Pharmacology, Pediatrics, and Gynecology and Obstetrics.

The module on Islam and family planning was interactive and encouraged discussion. Participants were asked at the beginning of the training session to list their own and their clients' perceptions about the permissibility of family planning in Islam. Misconceptions ranged from labeling family planning as a conspiracy to limit the size of the Muslim population to equating contraception with infanticide. Trainers used this list to provide counterarguments during the training, to help trainees address their own misconceptions, and to use the same arguments to convince others.

We also asked each participant to complete the following sentence: “For developing an ideal Islamic society, Muslim citizens should be __________.” Participants contributed such words as “strong,” “educated,” “healthy,” and “pious.”

We then asked participants what prerequisites they believed they had to fulfill to achieve these objectives. Responses consistently included: investing more in their children by giving them quality education, a proper upbringing, appropriate healthcare, and adequate time and attention. Participants argued that these requirements could be adequately fulfilled only if families spaced their births and had a manageable family size.

Next, we introduced the Quranic view on family wellbeing. Trainers explained that because the message of the Quran is for all times and that it provides its followers with a complete code of life, Muslims can seek guidance about contemporary issues by examining relevant principles laid down in the Quran.

Accordingly, trainers introduced participants to 6 specific verses of the Quran related to creating a healthy and pious family that have been cited in a number of materials on Islam and family planning.^11^-^12^

The starting point of the discussion was the Quranic verse that relates to spousal responsibility before entering matrimony:

*Let those who find not the wherewithal for marriage keep themselves chaste, until Allah gives them means out of His grace.*^16(24:33)^

This verse enjoins believers to enter into marriage *only* if one is capable of bearing the responsibility of raising a family and is in a position to meet the physical, social, and economic needs of the spouse and offspring. The explanation of this verse is that it is incumbent upon the husband to provide for the wellbeing and needs of his spouse and future offspring. If he is unable to do so, Allah advises him not to marry and to remain chaste until such time that Allah gives him the means to do so. The verse clearly outlines the concept of responsible parenthood, as marriage is not simply the union of individuals but also an institution for raising a family.

Next, participants discussed the meaning and interpretation of the following Quranic verse, which succinctly describes the attributes of a family:

*And those who pray, “Our Lord! Grant unto us wives and offspring who will be the comfort of our eyes, and give us (the grace) to lead the righteous.*^16(25:74)^

The Holy Quran enjoins Muslims to ask Allah for spouses and children that bring peace and tranquility to the heart. Children who bring “comfort to the eyes” of the parents are those who are undoubtedly healthy, educated, well-behaved, and devout Muslims. This requires a heavy investment of time and resources in the upbringing of children and meeting the requirements and needs of the spouse by providing them with the requisite amount of attention and care.

In this next verse, the Quran has equated possessions and progeny and labeled them as a source of trial in this world:

*And know ye that your possessions and your progeny are but a trial and that it is Allah with whom lies your highest reward.*^16(8:28)^

The Quran equates offspring and material possessions, describing both as a source of trial and tribulation. The lack of possessions and offspring can become a source of great anguish for parents. Similarly, excess wealth and offspring can be a source of trial as parents can become preoccupied with concerns and worries of how to protect and look after them and hence become distracted from following religious injunctions. A state of moderation and balance is therefore the ideal that one must pursue.

In the next 2 verses, the Quran lays down the principle of prolonged breastfeeding that physiologically leads to spacing between pregnancies—pointing to the permissibility and even encouragement of birth spacing.

Verses from the Quran encourage infant breastfeeding for at least 2 years—establishing the principle of prolonged breastfeeding that leads to birth spacing.

*The mothers shall give suck to their offspring (breastfeed) for two whole years.*^16(2:233)^

*In pain did his mother bear him, and in pain did she give him birth. The carrying of the (child) to his weaning is (a period of) thirty months.*^16(46:15)^

In these verses, the Quran is categorical about establishing the right of the child to be breastfed for at least 2 years. Medical science has established that breastfeeding has numerous health benefits in terms of enhancing children's immunity and protecting them from infections, apart from being a sterile source of nutrition. Furthermore, prolonged (exclusive) breastfeeding leads to lactational amenorrhea that prevents pregnancy, resulting in spacing between births. This, in turn, allows the mother to recover her strength after delivery and ensures that the offspring continues to receive adequate nutrition and maternal attention.

In the contraceptive technology training session, we discussed the Lactational Amenorrhea Method in detail and explained that the effectiveness of breastfeeding in suppressing ovulation (and preventing pregnancy) is reduced once weaning starts, at which point exogenous contraceptives are needed to prevent pregnancy.

We also showed the trainees a diagram that explained how exogenously administered hormonal contraceptives and breastfeeding follow the same mechanism acting on the hypothalamic pituitary pathway in the brain leading to a suppression of ovulation and prevention of pregnancy. We stressed that this is the principle that forms the basis of the permissibility of contraception in Islam.

The final verse discussed with the trainees relates to maternal health and wellbeing:

*Your women are lands (tilth) for you; so approach your tilth when or how you will.*^16(2:223)^

In this verse, the Quran has compared women's reproductive capability to the cultivable fields that produce food. All wise farmers know that they have to meet certain preconditions to obtain a good yield, such as providing nutrients to the soil, not sowing out of season, and giving gaps between cultivating the crops so that the soil can regain its productive capacity. Similarly, for families to reap healthy offspring, mothers' physiological needs have to be met in terms of providing proper diet and nutrients during and after pregnancy and allowing them to recoup their energy and strength lost during childbirth before becoming pregnant again. Through this metaphor, we can infer that the Quran once again reinforces the concept of birth spacing and also establishes women's rights to be respected and cared for.

Trainers also informed participants that Prophet Mohammad (peace be upon him, pbuh) never forbade Muslims to practice *al-'azl* (coitus interruptus, or the traditional family planning method of withdrawal), which was available to Muslims at that time.^17^^-^^18^As a result, the 5 major Islamic schools of thought (Hanafi, Maliki, Ja'fari [Imami], Hanbali, and Shafi) have permitted the practice of coitus interruptus.^14^^,^^19^

At this point, trainers synthesized the conversation and discussed some of the common arguments presented against family planning. For example, to counter the common argument that family planning is an attempt to check the growth of the Muslim population, trainers explained that the size of the Muslim community grew phenomenally after the advent of Islam not because of procreation but primarily because of the rapid conversions that took place, as a result of people being impressed by the message and pious conduct of the Prophet Mohammad (pbuh). Trainers also explained that family planning is not akin to infanticide, a practice explicitly prohibited in Islam, because it prevents the process that leads to the development of an infant.

Trainers also showed the video documentary of the endorsement of family planning by major religious scholars of Pakistan to further augment the arguments that had been discussed earlier. Trainers stressed that birth spacing helps save the lives of mothers and newborns. The trainers also provided participants with a list of edicts on Islam and family planning issued by Muslim scholars.

## EVALUATION METHODS

We conducted a situation analysis in 14 districts of 3 provinces of Pakistan (Khyber Pakhtunkhwa, Punjab, and Sindh) to assess the effect of the FALAH project, which included interviews with providers (both those who did and did not receive training). We randomly sampled 11 health facilities in each of the 14 districts and interviewed a total of 175 providers to assess their family planning knowledge. To assess the specific module on Islam and family planning, we held focus group discussions with providers before and after the training session to evaluate changes in perspectives and opinions regarding permissibility of family planning in Islam.

We also interviewed faculty members 2 years after the training to obtain their feedback on the overall training program, including on the Islam and family planning training module.

Finally, to measure impact of the FALAH project overall, which included several components to improve access to family planning, we conducted representative baseline and endline surveys of married women of reproductive age (ages 15 to 49) in 14 districts to measure changes in contraceptive prevalence and unmet family planning need. Security reasons prevented us from conducting the surveys in the remaining 6 project districts in the fourth province (Balochistan). In the 2008/09 baseline survey, we collected data from 10,604 women, and in the 2011/12 endline survey, from 12,403 women, through systematic random sampling of households. Each eligible married woman of reproductive age available in selected households was interviewed.

We submitted the evaluation proposal to the Institutional Review Board of the Population Council, which determined that the work met all requirements for informed consent and protection of confidentiality and that it was exempt from federal regulations because it represented minimal risk to the human subjects.

## RESULTS

The “Basic Minimum Family Planning Contents” training package with the module on Islam and family planning has been introduced to 174 faculty members of 6 major universities of Pakistan and their affiliated medical colleges: the University of Health Sciences Lahore; Dow University of Health Sciences Karachi; Khyber Medical University Peshawar; Shaheed Mohtarma Benazir Bhutto Medical University, Larkana; Peoples University of Medical and Health Sciences for Women Shaheed Benazirabad; and Liaquat University of Medical and Health Sciences, Jamshoro. The training module also has been incorporated into the teaching program conducted by the Population Welfare Program through its Regional Training Institutes all over Pakistan. This will undoubtedly ensure that existing as well as future providers continue to benefit from its contents.

### Perceptions of Medical College Faculty

Feedback from participants through follow-up interviews indicates that the module has helped medical college faculty address their own misconceptions and concerns that prevented them in the past from discussing the topic openly and candidly. Faculty members have also indicated that the module helped them provide appropriate responses to questions and arguments raised by students. Overall, the module has enhanced their level of confidence and made it easier to introduce the topic of family planning in a more convincing and non-controversial manner.

The module on Islam and family wellbeing has been most helpful as it allows us to answer queries of our students from a religious perspective. We explain that the concept of healthy timing and spacing of pregnancies is in consonance with Islamic teachings. We start our discussion by explaining the Lactational Amenorrhea Method and elaborate this by quoting Quranic verses. This greatly helps in establishing the permissibility of birth spacing and adoption of contraception in Islam.

—Head of Department of Community Medicine, Quaid-e-Azam Medical College Bahawalpur

Faculty members also appreciated the video with the endorsement of religious scholars, which provides an additional level of support to their arguments and clarifies students' misconceptions.

### Knowledge and Perceptions of Health Care Providers

More of the trained providers had an accurate understanding of the birth spacing concept per the WHO-recommended definition of HTSP (38%) than untrained providers (5%). In addition, more of the trained providers (78%) accurately explained family planning as a means to “plan your family according to your resources” compared with untrained providers (50%). Both concepts were covered in the module on Islam and family planning.

In addition, group discussions with trained providers indicated that the module on Islam and family planning had changed their perceptions about the permissibility of family planning.

Before attending the training, I viewed family planning as a sin … My views are now completely changed. I will now become an advocate for family planning.

—Senior Medical Officer, Jaffarabad, Balochistan

### Effects on Contraceptive Use

Although it is difficult to tease out the impact of the training component of the FALAH project through the situation analysis, overall we found that facilities with trained providers received, on average, 60% more family planning clients over a 6-month period prior to the assessment compared with facilities with untrained providers.^20^

In the 3.5 year-period of active program implementation, we found an overall increase of 9 percentage points in contraceptive prevalence in the program implementation districts—from about 29% to 38% (see Figure).^20^-^21^ We emphasize that this can in no way be attributed solely to the training as there were many additional inputs that facilitated clients' access to family planning services, yet the importance of the Islam and family planning module cannot be underestimated.

**Figure:**
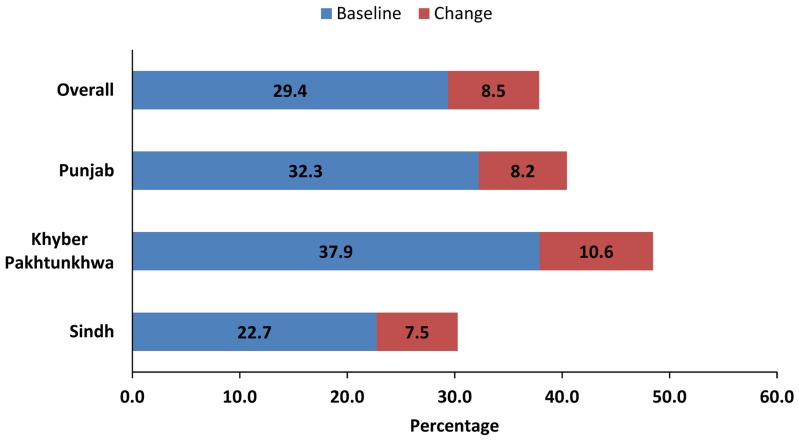
Change in Contraceptive Prevalence in FALAH Project Implementation Provinces Baseline survey among married women of reproductive age (ages 15–49) was conducted in 2008/09; endline survey in 2011/12. Surveys were not conducted in Balochistan province due to security reasons.

## DISCUSSION

Until recently, the public-sector family planning program in Pakistan did not make any serious attempt to seek the support of religious scholars in openly approving family planning. Other Muslim countries, such as Bangladesh, Egypt, Indonesia, Iran, and Tunisia, obtained endorsement from religious scholars much earlier, contributing to the success of their family planning programs. For instance:

- Morocco organized the Rabat Conference on Family Planning in 1971.
- Iran issued a national family planning policy in 1989 that the highest religious authorities had endorsed. Iranian religious leaders collaborated with health practitioners to promote family planning, using not only electronic and print media but also the pulpit of the mosques. In a span of 20 years, contraceptive prevalence in Iran rose from 37% to 73%.^15^,^22^-^23^
- Bangladesh made concerted efforts early on in its family planning program to educate Muslim clergy about the health rationale of family planning.^22^ Indonesia arranged a congress on Islam and Population Policy in 1990. Religious leaders have been invited to participate in seminars and workshops with the goal of increasing their family planning knowledge, influencing their attitudes about contraception, and motivating them to advocate family planning.^24^
- In a survey in Jordan, religious leaders who said that they believed family planning was acceptable in Islam registered significantly higher agreement scores (*P*<.001) on statements about the benefits of family planning than those who said that family planning was not allowed or who were uncertain.^25^ This suggested that religious leaders should not be ignored as potential proponents of family planning.
- Egypt obtained edicts supporting family planning from scholars at Al-Azhar University, the most reputed seat of Muslim learning, and Egypt's Grand Mufti publicly proclaimed that family planning was allowed in Islam.^26^

Our decision to include a module on the Islamic perspective on family planning in the training curricula for providers and medical faculty was indeed timely in not only removing misconceptions but also promoting family planning in Pakistan. Other countries that have sizeable Muslim populations with low rates of contraceptive prevalence could benefit from this module, particularly those countries that grapple with inhibitions to adopting family planning on religious grounds, such as Afghanistan, Chad, Mali, Mozambique, and Niger.
